# ESR Essentials: radiomics—practice recommendations by the European Society of Medical Imaging Informatics

**DOI:** 10.1007/s00330-024-11093-9

**Published:** 2024-10-25

**Authors:** João Santinha, Daniel Pinto dos Santos, Fabian Laqua, Jacob J. Visser, Kevin B. W. Groot Lipman, Matthias Dietzel, Michail E. Klontzas, Renato Cuocolo, Salvatore Gitto, Tugba Akinci D’Antonoli

**Affiliations:** 1 Digital Surgery LAB, Champalimaud Research, Champalimaud Foundation, Av. Brasília, 1400-038 Lisbon, Portugal; 2 Instituto Superior Técnico, Universidade de Lisboa, Av. Rovisco Pais 1, 1049-001 Lisbon, Portugal; 3 Department of Radiology, University Hospital of Cologne, Cologne, Germany; 4 Department of Radiology, University Hospital of Frankfurt, Frankfurt, Germany; 5 Department of Diagnostic and Interventional Radiology, University Hospital Wuerzburg, Wuerzburg, Germany; 6 Department of Radiology & Nuclear Medicine, Erasmus University Medical Centre, Rotterdam, The Netherlands; 7 Department of Radiology, The Netherlands Cancer Institute, Amsterdam, the Netherlands; 8 Department of Thoracic Oncology, Netherlands Cancer Institute, Amsterdam, the Netherlands; 9 Department of Radiology, University Hospital Erlangen, Maximiliansplatz 3, 91054 Erlangen, Germany; 10 Department of Radiology, School of Medicine, University of Crete, Heraklion, Crete, Greece; 11 Department of Medical Imaging, University Hospital of Heraklion, Crete, Greece; 12 Division of Radiology, Department of Clinical Science Intervention and Technology (CLINTEC), Karolinska Institute, Solna, Sweden; 13 Department of Medicine, Surgery and Dentistry, University of Salerno, Baronissi, Italy; 14 Dipartimento di Scienze Biomediche per la Salute, Università degli Studi di Milano, Milan, Italy; 15 IRCCS Istituto Ortopedico Galeazzi, Milan, Italy; 16 Institute of Radiology and Nuclear Medicine, Cantonal Hospital Baselland, Liestal, Switzerland

**Keywords:** Radiomics, Image pre-processing, Feature extraction, Radiomics model training and assessment, Reproducibility

## Abstract

**Abstract:**

Radiomics is a method to extract detailed information from diagnostic images that cannot be perceived by the naked eye. Although radiomics research carries great potential to improve clinical decision-making, its inherent methodological complexities make it difficult to comprehend every step of the analysis, often causing reproducibility and generalizability issues that hinder clinical adoption. Critical steps in the radiomics analysis and model development pipeline—such as image, application of image filters, and selection of feature extraction parameters—can greatly affect the values of radiomic features. Moreover, common errors in data partitioning, model comparison, fine-tuning, assessment, and calibration can reduce reproducibility and impede clinical translation. Clinical adoption of radiomics also requires a deep understanding of model explainability and the development of intuitive interpretations of radiomic features. To address these challenges, it is essential for radiomics model developers and clinicians to be well-versed in current best practices. Proper knowledge and application of these practices is crucial for accurate radiomics feature extraction, robust model development, and thorough assessment, ultimately increasing reproducibility, generalizability, and the likelihood of successful clinical translation. In this article, we have provided researchers with our recommendations along with practical examples to facilitate good research practices in radiomics.

**Key Points:**

- *Radiomics’ inherent methodological complexity should be understood to ensure rigorous radiomic model development to improve clinical decision-making*.
- *Adherence to radiomics-specific checklists and quality assessment tools ensures methodological rigor*.
- *Use of standardized radiomics tools and best practices enhances clinical translation of radiomics models*.

## Key recommendations

- Radiomics research offers the opportunity to improve clinical decision-making (Level of evidence: Moderate) [1], but researchers should understand its inherent methodological complexity to ensure rigorous radiomic model development and smooth clinical implementation (Level of evidence: High).
- Researchers should adhere to radiomics-specific checklists, such as the CheckList for EvaluAtion of Radiomics research (CLEAR), and use quality assessment tools, such as the METhodological RadiomICs Score (METRICS) when conducting radiomics research (Level of evidence: Low).
- Radiomics feature extraction should be performed with standardized tools that comply with the Image Biomarker Standardisation Initiative (IBSI) guidelines, such as PyRadiomics, and researchers should adhere to currently known best practices to increase clinical translation (Level of evidence: Moderate).

## Introduction

Radiomics holds great promise in improving clinical decision-making, patient management, and clinical outcomes. Alas, as we progress through the second decade of radiomic research, clinical implementation of this promising method still lags far behind [2].

Radiomics is limited by methodological complexity. This is why it remains challenging to reproduce and implement published models in real-life clinical practice. Guidelines [3] and assessment tools [4] are available and aid both researchers and clinicians in conducting high-quality radiomic research. Nevertheless, finding suitable support for conducting valuable research in radiomics remains a challenge.

**Fig. 1:**
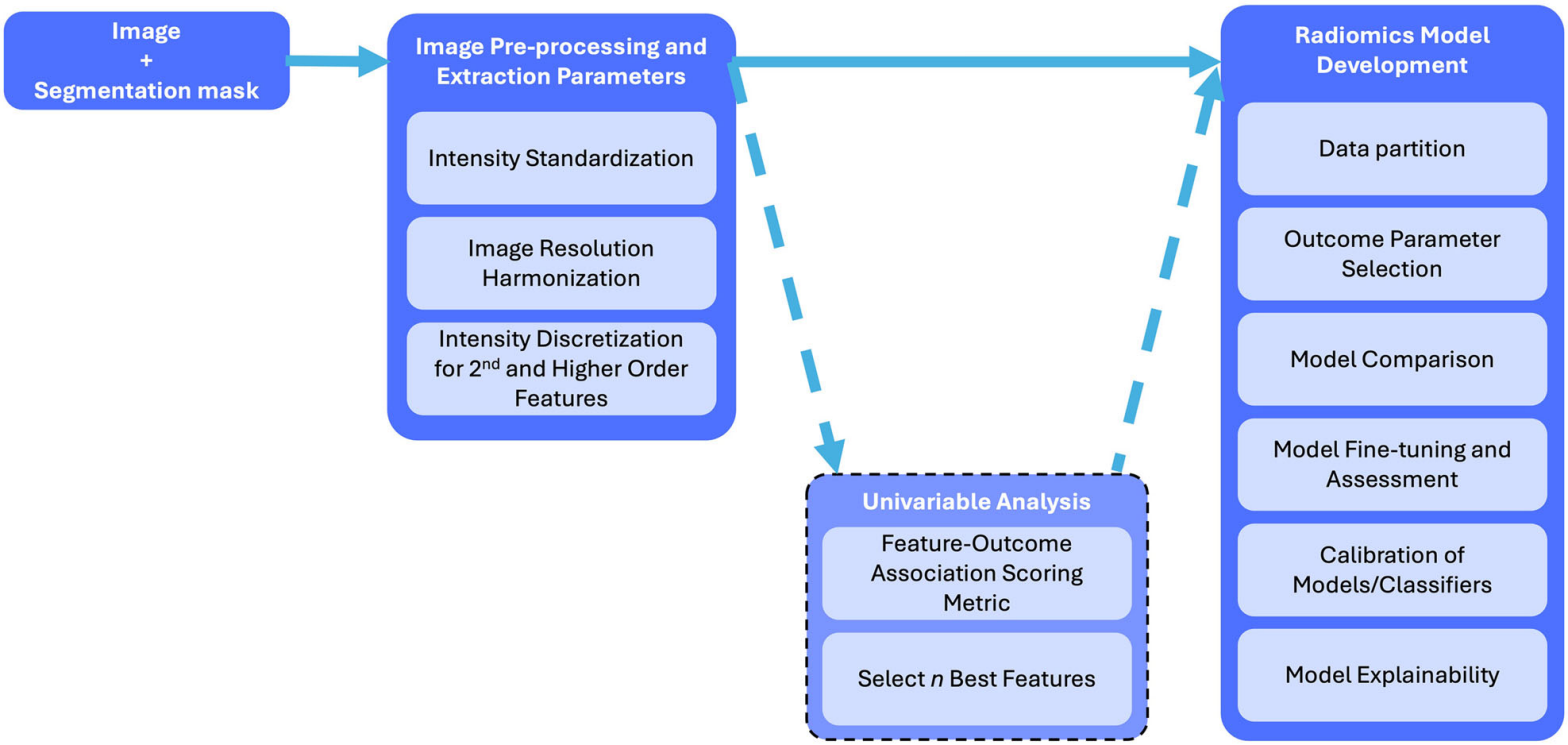
Flowchart summarizing the steps required to obtain a Radiomics Model, developed using best practices recommendations

As medical imaging continues to advance, mastering the complexity of radiomic methodology requires not only theoretical understanding but also the guidance of practical examples. Based on practical examples we aim to provide clinicians, researchers, and data scientists a roadmap to unlock the complex methodology of radiomics and increase reproducibility, generalizability, and ultimately clinical adoption. Hereby we utilize real-world examples and outline key steps of the radiomic workflow. We cover the entire pipeline, starting from image pre-processing, feature interpretability, and model development to model evaluation, as shown in Fig. 1. By emphasizing practical examples, we aim to empower readers to understand the challenges of the method and to confidently apply radiomics in their research. At the end of each section, we provide a link to the corresponding notebook. Here you can try out the main teaching points by yourself, using the ProstateX and respective public lesion segmentation datasets [5, 6], and eventually may customize the given items for your own research. These notebooks are also available at https://github.com/JoaoSantinha/RadiomicsEurRadEssentialsPaper.

## Radiomics features: extraction parameters, image pre-processing, filters for higher-order features and intuitions for interpretability

In this section, we review the best practices related to radiomics features, from feature extraction through image pre-processing, image filters, and feature interpretability.

### Extraction parameters and image pre-processing

In the radiomics workflow, image pre-processing is performed to standardize image quality, circumvent acquisition susceptibilities, and ensure the reproducibility of radiomic features [7]. Intensity normalization is one of the most important image pre-processing steps [8].

In cases where voxel intensities have units (e.g., CT-HU, PET-SUV, MRI-ADC-mm^2^/s, etc.), we can expect some degree of standardization of the intensities across patients. However, in qualitative imaging (e.g., non-quantitative MRI), the intensity units are arbitrary. In this scenario, the intensity of a tissue is related to the tissue type, but relative to the neighboring tissues represented in the image. As such, normalization methods like z-score normalization, WhiteStripe [9], Min-Max normalization, and histogram-based techniques [10] such as histogram matching and Contrast Limited Adaptive Histogram Equalization (CLAHE) [11] may be needed to standardize the intensities according to the subject, exam, and imaging equipment.

The open-source Python package PyRadiomics is the most common framework for radiomics feature extraction [12]. It adheres to the Image Biomarker Standardisation Initiative (IBSI) guidelines, having the differences well-described in PyRadiomics documentation (https://pyradiomics.readthedocs.io/en/latest/faq.html?highlight=IBSI#does-pyradiomics-adhere-to-ibsi-definitions-of-features), and, as in other frameworks, images and corresponding segmentations are used to extract radiomics features for further analysis. Such extraction relies on the fine-tuning of several parameters to ensure meaningful feature values. These parameters include intensity discretization, voxel size resampling, etc., and the reporting of such parameters is imperative in any radiomics manuscript to ensure reproducibility [13].

Intensity discretization enhances noise reduction and improves the reproducibility of the extracted features [14]. Bin width (each intensity bin will have this predefined width) is often favored over bin count (image intensities are stretched or squeezed so that the intensities within the mask will be divided into this predefined number of bins) due to its independence from the intensity range in the selected segment, which reduces segmentation variability [15]. This approach yields a higher number of reproducible radiomics features [16, 17]. A comprehensive phantom study attempted to find an optimal bin width that could result in the highest reproducibility of radiomic features [12]. However, although a certain degree of variability in feature values was reported for different bin widths, the authors could not demonstrate that feature stability was greatly influenced by the choice of bin width and could not indicate a certain bin width as being most optimal. Therefore, consistent reporting of all pre-processing steps was recommended to improve reproducibility [12]. The selection of bin width depends on the intensity range in the areas of interest. As no clear guidelines are currently available, it is advised to choose a bin width resulting in a number of bins between 16 and 128, which showed good reproducibility for fixed bin count with no significant differences in features computed within this range [18, 19].

Image resolution is also of major importance when dealing with a dataset exhibiting voxel spacing heterogeneity, as standardization through resampling is crucial to enhance the reproducibility of radiomics features [20]. This ensures that differences in radiomic features do not solely arise from resolution differences. It is important to determine whether images are isotropic or anisotropic and choose the resample voxel size accordingly, considering characteristics like in-plane resolution and slice thickness.

Even though isotropic images can be obtained from anisotropic ones by downsampling in-plane resolution (with information loss) or upsampling through-plane resolution (often not recommended due to the use of non-acquired information), a safer approach involves resampling in-plane resolution and extracting features in 2D instead of 3D, typically used in isotropic images.

### Image filters for higher-order radiomics features

**Fig. 2:**
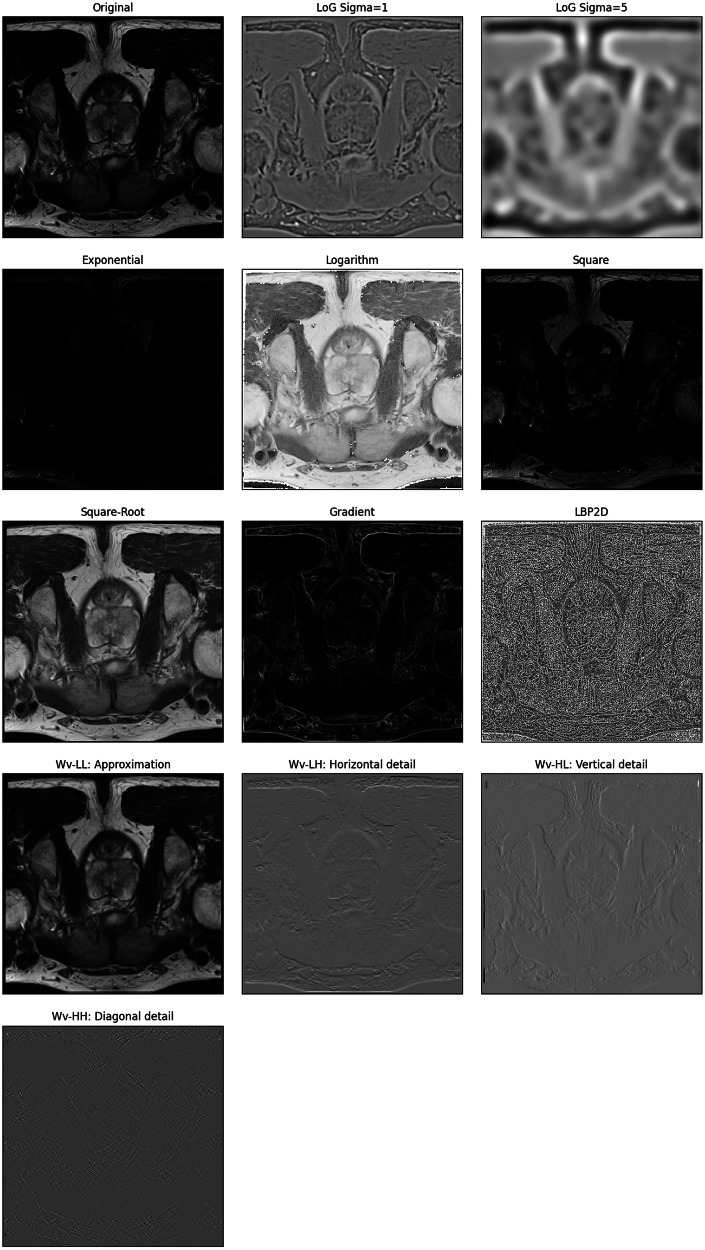
Example of common filters applied to the images

Features are critical for achieving high predictive performances in radiomic studies, and pre-processing filters (e.g., Laplacian of Gaussian, Wavelets, Exponential, Logarithm, Square, Square-Root, etc.) can be applied to images before feature extraction to enhance quantification of clinically relevant characteristics and patterns in medical images [21]. Examples of the output of these filters are shown in Fig. 2. The Image Biomarker Standardization Initiative recently introduced a standardized set of imaging modality-independent convolutional filters (e.g., Mean, Laplacian-of-Gaussian, Laws and Gabor kernels, separable and non-separable wavelets—including decomposed forms, and Riesz transformations) shown to enhance reproducibility [21].

While filters expand the number of texture features to several hundred or even thousands, potentially complicating the creation of interpretable radiomic models—particularly in studies with a limited number of cases—recent findings by Demircioglu [22] showed that combining features extracted from original and filtered images may be beneficial, as this approach yielded similar or even superior predictive performance compared to using only original image features across various public image datasets.

In the following Google Colab notebook link, we illustrate the results of applying each of the filters to the original image (https://colab.research.google.com/github/JoaoSantinha/RadiomicsEurRadEssentialsPaper/blob/main/Image%20and%20Filtered%20Images.ipynb).

### Intuitions for radiomics feature interpretability

Interpretability stands out as a critical barrier hindering the integration of radiomics analysis into clinical practice. The complexity of features directly impacts their interpretability; while first-order features and shape-related metrics are relatively straightforward, textural features and those derived from image filtering pose greater challenges.

**Fig. 3:**
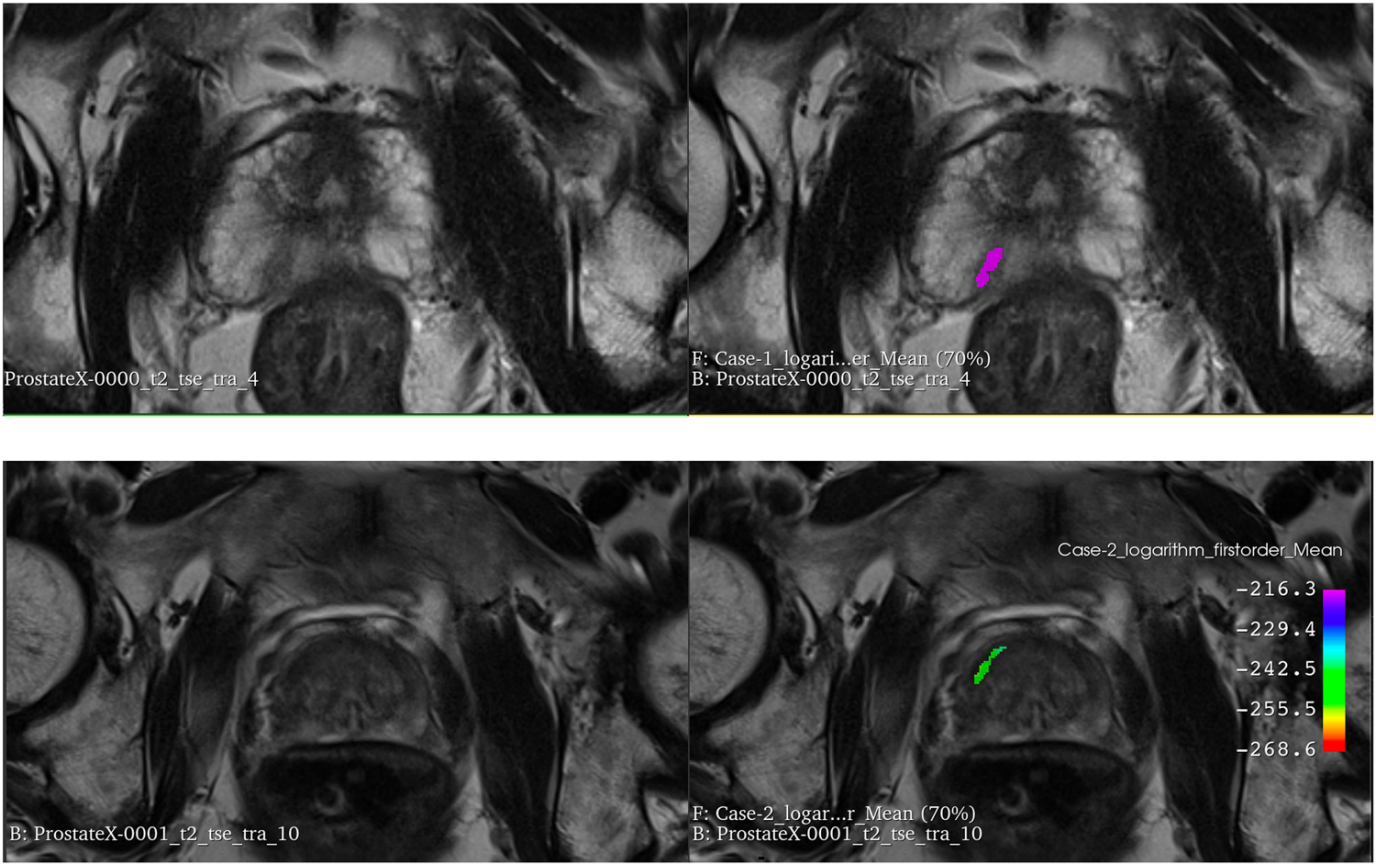
Radiomic maps of logarithm_firstorder_Mean feature for a positive case (two images at the top, corresponding to finding 1 of ProstateX-0000) and a negative case (two images at the bottom, corresponding to finding 1 of ProstateX-0001). The images on the left are the T2w images without overlays, and the images on the right represent the logarithm_firstorder_Mean radiomic maps of the respective findings overlaid to the respective T2w images (equal windowed PET clut was used for right top and bottom overlays)

While employing transparent methodologies and adhering to standardized radiomics checklists, such as CheckList for EvaluAtion of Radiomics research (CLEAR) [3], and quality assessment tools, such as METhodological RadiomICs Score (METRICS) [4], is essential, interpretability issues must also be addressed [23]. As such, efforts must be made to correlate radiomics features with biological variables to enhance trust in the methodology. Additionally, further efforts must be made to correlate radiomics features with biological variables to enhance trust in the methodology. For instance, certain features like entropy have direct correlations with biological parameters such as tumor heterogeneity [24]. Utilizing color mapping to visualize features in relation to tissue types of interest, as shown in Fig. 3, further improves interpretability and facilitates clinical adoption [25].

## Radiomics feature univariable analysis

Univariable analyses allow assessing association of each radiomic feature with the outcome of interest [25]. To assess such associations, methods like the area under the receiver operating characteristic curve [26, 27], Pearson’s correlation test [28], chi-square test, *t*-test, Welch’s *t*-test, Mann–Whitney *U*-test, variance, Relief, and mutual information can be used [29]. Given the challenge posed by a large number of features and relatively small datasets, these methods often serve as feature selection tools, aiming to reduce dimensionality and prevent overfitting, aligning with the “purposeful selection” approach advocated by Hosmer and Lemenshow [30–32]. Typically, statistical significance (often indicated by *p* < 0.05) guides feature inclusion in multivariate modeling [33], accompanied by multiple comparison corrections [34, 35]. Additionally, unsupervised clustering has been used to select a single representative feature from each cluster for use in modeling [36].

Despite its common usage in radiomics and other medical sciences, univariable analysis-based feature selection may be considered inappropriate as it may produce inaccurate determinations of the contributions of radiomic features to outcomes [33].

Another commonly observed approach in radiomics studies involves the criterion of *n* patients per radiomic variable for multivariable analysis, which has shown weak evidence for binary logistic regression analysis [37] and where shrinkage methods may be better suited to perform feature selection as part of the model fitting procedure and reduce model overfitting [38].

The following Google Colab notebook shows an example of univariable analysis and subsequent use of its information for modeling, comparing it with a radiomics model developed using best practices (https://colab.research.google.com/github/JoaoSantinha/RadiomicsEurRadEssentialsPaper/blob/main/Radiomics%20Features%20Univariable%20Analysis.ipynb).

## Best practices for radiomics model development

In this section, we review best practices related to several steps of model development and assessment, where mistakes commonly jeopardize the developed models.

### Data partitioning

Correct data partitioning is essential to avoid information leakage and to avoid biasing the training process by providing information from test data [3]. Therefore, it is imperative that the data is split on a patient level. Hyperparameter optimization can be performed using validation data, acting as a preliminary benchmark, to be confirmed with subsequent testing of the final model.

Validation data can be obtained from a single partitioning (hold-out), but this method is prone to sampling issues. Resampling methods are therefore preferable, e.g., bootstrapping or k-fold cross-validation (CV). CV can take several forms, based on label distribution (stratified CV), with inner and outer loops (nested CV), or allow case-by-case inference (leave-one-out CV) [39]. Results can be averaged across multiple iterations by using different seeds.

Once the pipeline-model configuration is finalized, confirming validation results requires a novel test sample. Ideally, collecting new patient data post-model development offers the most robust validation approach. In retrospective studies, it is common practice to reserve a portion of the initial data. However, to avoid random sampling issues, a “temporal” splitting technique is preferred, which requires acquiring new patients after model development [40].

### Outcome parameter selection

Selecting a (relevant) outcome parameter(s) demands a systematic approach, which considers the stage of the radiomics model. The Radiology AI Deployment and Assessment Rubric (RADAR) model provides a structured framework for this purpose [41], drawing from Fryback and Thornbury’s diagnostic efficacy framework [42]. An important concept within this framework is that demonstrating value in the pre-clinical phase does not guarantee the same in the clinical phase.

The pre-clinical phase evaluates technical efficacy and diagnostic accuracy. Technical efficacy assesses the model’s ability to process relevant images, while diagnostic accuracy concerns its sensitivity and specificity. Moving to the clinical phase, the model’s actual impact on clinical care is evaluated. Parameters such as its influence on diagnostic reasoning, treatment decisions, and patient outcomes are assessed. Furthermore, health economic evaluations are imperative to determine the cost-effectiveness of implementing the radiomics model, as illustrated by a recent study on the clinical and economic impact of integrating a radiogenomics model with clinical data in identifying BRCA mutation carriers in the general population [43].

### Model comparison

After extracting features and partitioning the data, various models can be constructed using the radiomic features as input [44]. Depending on the specific target of interest, a range of model classes can be employed, including but not limited to regression models, tree-based models, neural networks, or support vector machines. However, a single evaluation metric is insufficient for a comprehensive assessment when comparing different model types.

To understand the intricacies of different models, several statistical tests are available to evaluate clinically relevant performance metrics [45]. For classification tasks, tests like the McNemar test and Cochran’s Q test for confusion matrices, DeLong test for area under the curve, or F-test for variances can be employed to compare models. These tests should be applied to models that have been trained and evaluated using the same process (e.g., hold-out, cross-validation, stratified cross-validation, nested cross-validation, or others) to ensure that differences are not due to varying procedures. Subsequently, researchers should weigh the impact of false positives and false negatives independently, considering the consequences of each type of mistake. In certain clinical contexts, false negatives may have more severe repercussions for patients than false positives. Therefore, thoughtful consideration is essential when comparing models and their respective performance metrics [46].

### Model fine-tuning and assessment

Hyperparameter optimization and fine-tuning are crucial for maximizing model performance. Since radiomic features are non-learnable hard-coded, the learning occurs upon the extracted features [12], facilitating computationally cheap ‘hyperparameter sweeps’, where the researcher tests many hyperparameter setups to reach optimal performance, when compared to deep learning models.

A common error is repeatedly testing the model on the test set, leading to test set overfitting [47]. Therefore, optimization must exclusively occur on the validation set. Once the best model with respective parameters is identified, the test set should be used once to report the final performance metrics. To increase the generalizability of results from validation to test scores, a relatively large and diverse validation set is recommended for hyperparameters sweeps.

### Calibration of models/classifier

Machine learning models output scores between 0 and 1 that are often uncalibrated, meaning they do not accurately reflect the likelihood of aligning with the reference standard. For example, if a model consistently scores cases at 0.6 but is correct 90% of the time, one would expect the scores to reflect this accuracy, perhaps as 0.9. Calibration aims to rectify these discrepancies [45].

Various calibration methods try to mitigate these misalignments. One example is Platt’s scaling, yet it assumes a logistic relationship, which may not always hold. A more recent approach gaining traction is conformal prediction [48], which requires a separate hold-out set for calibration and provides mathematical assurances of score-probability alignment. Subsequently, calibration curves can be generated to assess the expected calibration error (ECE), indicating the degree to which output scores align with probabilities. Moreover, it is essential to examine the histogram of output score bins against underlying probabilities to ensure the distribution is not significantly skewed toward 0 or 1.

### Model explainability

**Fig. 4:**
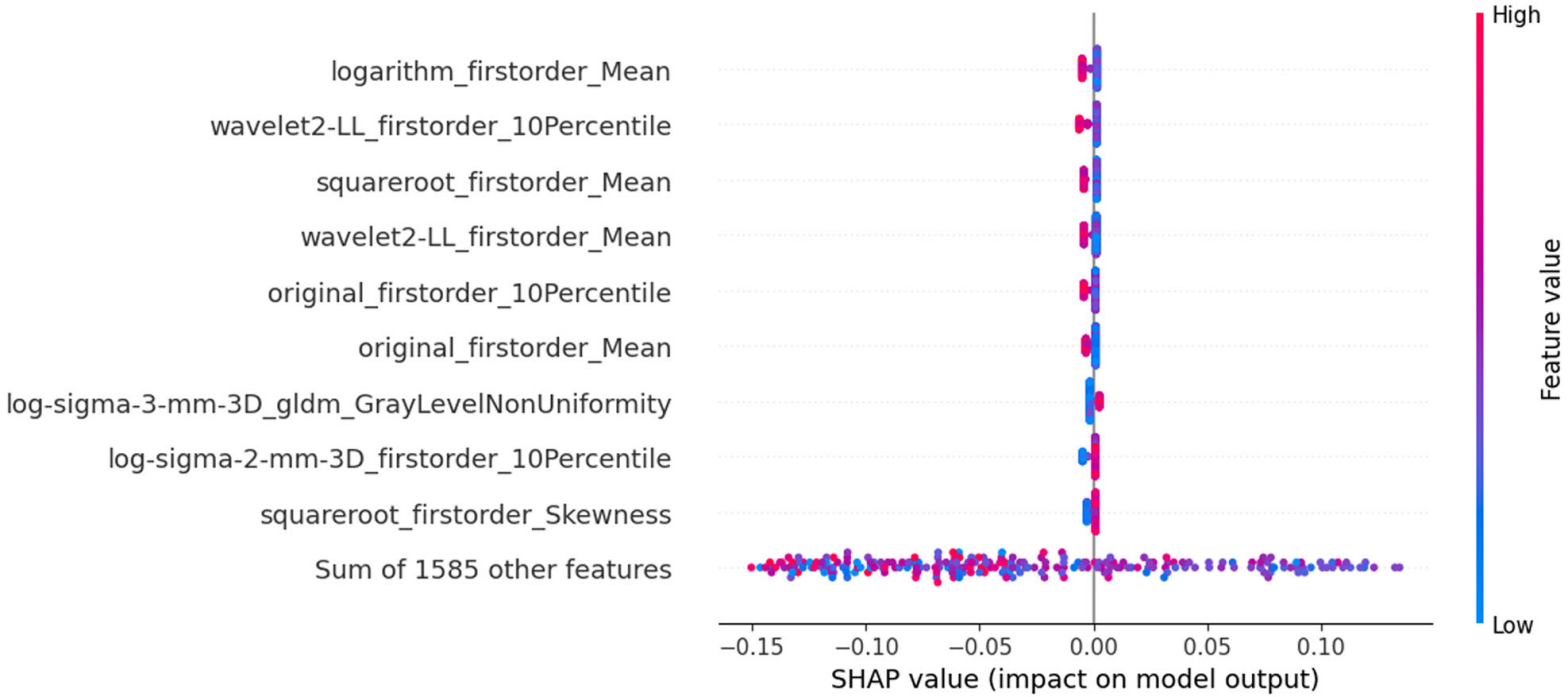
Example of SHAP analysis summary output for a Random Forest Classifier trained with a dataset used in Jupyter Notebooks

When developing models, there’s typically a trade-off between explainability and performance. Linear classifiers, for instance, offer explainability but might be outperformed by more complex machine learning models. Therefore, achieving both explainability and performance is an active area of research in Explainable AI. Clinicians and researchers must assess each clinical use-case to determine the required level of explainability and consider the model’s performance relative to its complexity. Statistical measures like Akaike Information Criteria (AIC) can aid in optimal decision-making in this regard. Furthermore, conducting in-depth analysis and reasoning enables the formulation of hypotheses about the model’s behavior and logical extraction of explainability [49]. Methods like the SHapley Additive exPlanations (SHAP) [50], e.g., Fig. 4, and Local Interpretable Model-agnostic Explanations (LIME) [51] have been widely used for model explainability.

In the next Google Colab notebook, we will go through all aspects of model development here mentioned (https://colab.research.google.com/github/JoaoSantinha/RadiomicsEurRadEssentialsPaper/blob/main/Radiomics%20Features%20Model%20Development.ipynb).

### Open science

Last but certainly not least, is the open science status of several aspects related to the development of a radiomics model. These essential open science items include the public availability of data, parameter files, code, and model, and their availability will result in clarity in methodological steps, transparency, and reproducible research [2, 52].

## Summary statement

In this comprehensive tutorial, we provide essential knowledge to master the complexity of the entire radiomic pipeline. Following the life cycle of radiomics research, we start from image pre-processing and travel through the entire radiomics pipeline up to model explainability (Table 1 and Fig. 1).

**Table 1:** Summary of recommendations for best practices in radiomics research

Radiomics analysis	Main steps	Recommendation	Notebook link
**Features**	Image pre-processing	Harmonize voxel intensities if needed. Ensure that image resolution is standardized across patients.	https://colab.research.google.com/github/JoaoSantinha/RadiomicsEurRadEssentialsPaper/blob/main/Image%20Pre-processing.ipynb
	Feature extraction parameters	Choose bin-width or bin-count texture features with intensity discretization. For bin-width, select a value within the range of 16 to 128 across patients.	https://colab.research.google.com/github/JoaoSantinha/RadiomicsEurRadEssentialsPaper/blob/main/Radiomics%20Feature%20Extraction.ipynb
	Image filters for higher-order radiomic features	Apply imaging modality-independent convolutional filters shown to enhance reproducibility.	https://colab.research.google.com/github/JoaoSantinha/RadiomicsEurRadEssentialsPaper/blob/main/Image%20and%20Filtered%20Images.ipynb
	Intuitions for features interpretability	Employ transparent methodologies and adhere to standardized radiomics guidelines, such as CLEAR. Extract radiomic maps of model’s most important features and use color mapping to improve interpretability.	https://colab.research.google.com/github/JoaoSantinha/RadiomicsEurRadEssentialsPaper/blob/main/Radiomics%20Features%20Model%20Development.ipynb#scrollTo=j_n8HbSoEvKp
	Univariable analysis	Use univariable analysis to assess each radiomic feature’s association with the outcome of interest. Keep in mind that there is weak evidence for the criterion of n patients per radiomic variable in multivariable binary logistic regression analysis.	https://colab.research.google.com/github/JoaoSantinha/RadiomicsEurRadEssentialsPaper/blob/main/Radiomics%20Features%20Univariable%20Analysis.ipynb
**Model development**	Data partitioning	Split data on a patient level. Validation data can be obtained from a single partitioning (hold-out) or using resampling methods such as bootstrapping or k-fold cross-validation (CV), stratified CV, nested CV, or leave-one-out CV. Apply “Temporal” splitting to avoid random sampling issues and simulate acquiring new patients after model development.	https://colab.research.google.com/github/JoaoSantinha/RadiomicsEurRadEssentialsPaper/blob/main/Radiomics%20Features%20Model%20Development.ipynb#scrollTo=s19f8hIYEvKb
	Outcome parameter selection	Follow the Radiology AI Deployment and Assessment Rubric (RADAR), a structured framework for selecting a (relevant) outcome parameter(s). Evaluate the cost-effectiveness of implementing the radiomics model.	Not applicable
	Model comparison	Use several evaluation metrics to comprehensively assess different models. Weight the impact of false positives and false negatives independently, and consider the consequences of each type of mistake in the specific clinical context.	https://colab.research.google.com/github/JoaoSantinha/RadiomicsEurRadEssentialsPaper/blob/main/Radiomics%20Features%20Model%20Development.ipynb#scrollTo=o5g5FPdWEvKe&line=1&uniqifier=1
	Model fine-tuning and assessment	Apply hyperparameter optimization techniques to models using non-learnable hard-coded radiomic features. Optimization must exclusively occur on the validation set. Avoid test set overfitting, by using the test set only once to report the final performance metrics of the best model.	https://colab.research.google.com/github/JoaoSantinha/RadiomicsEurRadEssentialsPaper/blob/main/Radiomics%20Features%20Model%20Development.ipynb#scrollTo=DWZBFVkAfjps
	Calibration of models/classifier	Use calibration methods, such as Platt’s scaling and conformal prediction, to mitigate model misalignment. Use calibration curves to assess the expected calibration error (ECE).	https://colab.research.google.com/github/JoaoSantinha/RadiomicsEurRadEssentialsPaper/blob/main/Radiomics%20Features%20Model%20Development.ipynb#scrollTo=saqTdCnEEvKi&line=3&uniqifier=1
	Model explainability	Use methods such as Akaike Information Criteria (AIC) to aid in optimal decision-making and to determine the required level of explainability and desired model’s performance relative to its complexity. Evaluate approaches such as SHAP and LIME to explain multivariable models.	https://colab.research.google.com/github/JoaoSantinha/RadiomicsEurRadEssentialsPaper/blob/main/Radiomics%20Features%20Model%20Development.ipynb#scrollTo=pb6Ff8M2EvKk

“Radiomics Feature Extraction and Pre-processing” highlights the importance of standardizing images across different settings and modalities, thereby achieving more reproducible results. “Univariable Analysis” explores the association of radiomics features with clinical outcomes; thereby, it supports the pivotal step of “feature selection” and outlines the potential pitfalls to be considered. If done appropriately, univariate analysis reduces overfitting and supports the development of robust radiomics models.

“Radiomics Model Development” requires meticulous data partitioning and outcome parameter selection, which might be supported by applying the RADAR framework. To validate radiomics results, validation strategies have to be formalized. Using a hold-out test set is crucial for developing robust radiomic models. Following these approaches can help understand how well models actually perform in real-world situations. Different statistical tests are available for “Model Comparison”. It is essential to avoid overfitting, which can be a result of repeated testing of the model during model fine-tuning. Calibration methods are recommended to align model outputs to real-world scenarios.

“Model explainability” is key in the development of meaningful radiomic models. Although simpler models often provide better interpretability, they may come at the expense of model performance. Explainability plays a crucial role in the implementation of radiomic models in the clinical setting, and adherence to radiomic-specific guidelines will help researchers achieve explainability.

### Patient summary

Radiomics is a promising tool for analyzing medical images. In this article, we provide a comprehensive overview of the underlying methodology and investigate all essential steps of the radiomics pipeline, which include pre-processing, univariate analysis, data partitioning, outcome parameter selection, model evaluation, and development of interpretable radiomic models. We also discuss methodological challenges regarding feature selection, data handling, selection of outcome parameters, and overfitting.

## Abbreviations

AIC: Akaike Information Criteria
CLEAR: CheckList for EvaluAtion of Radiomics research
CV: Cross-Validation
ECE: Expected Calibration Error
IBSI: Image Biomarker Standardisation Initiative
LIME: Local Interpretable Model-agnostic Explanations
METRICS: METhodological RadiomICs Score
RADAR: Radiology AI Deployment and Assessment Rubric
SHAP: SHapley Additive exPlanations
